# Single Amino Acid Supplementation in Inherited Metabolic Disorders: An Evidence-Based Review of Interventions

**DOI:** 10.3390/genes16050502

**Published:** 2025-04-27

**Authors:** Elvira Verduci, Martina Tosi, Carlo Dionisi Vici, Marco Spada

**Affiliations:** 1 Department of Health Sciences, University of Milan, 20142 Milan, Italy; martina.tosi@unimi.it; 2 Metabolic Diseases Unit, Department of Pediatrics, Vittore Buzzi Children’s Hospital, 20154 Milan, Italy; 3 Department of Pediatrics, Vittore Buzzi Children’s Hospital, 20154 Milan, Italy; 4 Division of Metabolic Diseases and Hepatology, Bambino Gesù Children’s Hospital IRCCS, 00165 Rome, Italy; carlo.dionisivici@opbg.net; 5 Department of Pediatrics, University of Torino, 10124 Torino, Italy; marco.spada@unito.it

**Keywords:** inherited metabolic disorders, amino acids, supplementation, branched-chain amino acids, nutrition

## Abstract

Background/Objectives: Inherited metabolic disorders (IMDs) are a group of genetic conditions affecting metabolic pathways. The treatment of some IMDs requires the dietary restriction of specific amino acids. IMDs may also necessitate the supplementation of one or more amino acids due to factors such as reduced dietary intake, impaired synthesis, defective transport or absorption, or increased utilization. This literature review aims to evaluate the most recent evidence regarding amino acid supplementation in IMDs, considering not only the prevention of amino acid deficiency and toxic accumulation but also the competition with other toxic metabolites. Methods: A systematic search strategy was developed and applied to PubMed/Medline and Scopus databases to identify relevant studies. Amino acids were categorized into six groups: branched-chain amino acids, aromatic amino acids, sulfur amino acids, urea cycle amino acids, other essential amino acids, and other non-essential amino acids. Results: A total of 24 rare IMDs were evaluated. A final number of 99 selected articles were assessed based on the Oxford Centre for Evidence-Based Medicine 2011 Levels of Evidence. Although this work represents a preliminary non-systematic review, it highlights the need for further studies and data collection. Conclusions: Future research must establish the plasma amino acid levels that indicate the need for supplementation, specify the appropriate dosages (g/day or mg/kg/day), determine the optimal treatment duration, and, crucially, define the target plasma ranges to be maintained for effective management of IMDs.

## 1. Introduction

Inherited metabolic disorders (IMDs) are a group of rare genetic disorders caused by defects in the genes encoding enzymes, transporters, receptors, and signaling molecules involved in metabolic processes. The most recent international classification of inherited metabolic disorders (ICIMD) includes over 1450 disorders [1]. A simplified classification categorizes IMDs into three main groups: (a) small molecule disorders, (b) complex molecule disorders, and (c) disorders affecting energy metabolism [2]. Small-molecule disorders can be subdivided into two functional categories: the “intoxication” type, characterized by acute or chronic accumulation of toxic compounds upstream of the metabolic block, and the deficiency type, resulting from defective synthesis or transport of compounds downstream of the metabolic block. Both categories include disorders affecting amino acid synthesis and degradation, organic acid metabolism, the urea cycle, and the metabolism of vitamins and other essential cofactors, and their treatment relies on lifelong nutritional interventions to prevent the toxic accumulation of substrates and metabolites or to restore deficiencies in enzymatic products [3].

Amino acids (AAs) are organic compounds that contain both an amino and a carboxyl group, along with a specific side chain that defines their properties. Amino acids are classified into three main groups: essential amino acids (e.g., isoleucine, leucine, valine, lysine, methionine, phenylalanine, threonine, tryptophan, and histidine), which cannot be synthesized by the body; conditionally essential (or semi-essential) amino acids, which may not be synthesized in sufficient amounts under specific conditions; and non-essential amino acids [4]. AAs play a crucial role in protein synthesis, tissue repair, and muscle regeneration, particularly after injuries or intense physical activity. They also contribute to the production of enzymes, proteins, hormones, and antibodies, supporting metabolism and immune function. AAs are found in both animal-derived foods (e.g., meat, fish, eggs, and dairy) and plant-based sources (e.g., pulses, grains, nuts, and seeds). Proteins from animal-derived foods have higher digestibility than plant-based proteins, which typically contain reduced quantities of one or more essential amino acids relative to proteins of higher biological value [5] (Supplementary Table S1).

The three branched-chain amino acids (BCAAs), leucine (Leu), isoleucine (Ile), and valine (Val), are essential amino acids involved in key metabolic processes, including skeletal muscle metabolism, the activity of branched-chain keto acid dehydrogenase (BCKD), and the amination of branched-chain keto acids (BCKAs) to BCAAs [6]. Recent studies have shown that elevated circulating BCAAs levels are associated with a higher risk of cardiometabolic diseases, including obesity, diabetes, and cardiovascular diseases [7]. In fact, elevated plasma BCAA concentrations can enhance Leu’s role as a stimulator of insulin secretion [8], which suppresses the oxidation of fatty acids [9], and may promote the accumulation of fat mass, establishing a link between a higher body mass index (BMI) and a higher protein intake. Supplementary Material S1 provides an overview of the metabolic role of amino acids in physiological and clinical contexts, while Supplementary Figure S1 illustrates the relationship between high protein and BCAA intake with the nutritional-metabolic impact on insulin and fatty acid metabolism.

In many IMDs, most of which belong to the category of small-molecule disorders, treatment may require supplementation with single amino acids. The rationale for supplementing a single amino acid may depend on the need to compensate for a deficiency, compete with the absorption or transport of other harmful amino acids, or provide an activating substrate for malfunctioning metabolic or cellular pathways. Most often, single amino acid supplementation is required to correct deficiencies caused by reduced nutritional intake, impaired synthesis, decreased absorption or transport, or increased consumption. To date, only one systematic literature review [10] has explored these aspects in certain aminoacidopathies.

This review aims to examine the role of single amino acid supplementation by evaluating the current evidence on its efficacy in preventing deficiencies and toxic accumulation and in modulating competition with other toxic metabolites. The focus is on a field where scientific data remain limited or inconclusive and where established guidelines or strong recommendations are currently lacking.

## 2. Materials and Methods

This narrative review was conducted with a systematic review approach following the PRISMA guidelines (Preferred Reporting Items for Systematic Reviews and Meta-Analysis) statement [11].

### 2.1. Data Sources and Search Strategies

Firstly, a panel of Italian IMD experts was convened to identify the relevant amino acids to be included in the research, focusing on amino acids and conditions with the most relevant clinical significance. Subsequently, a PICO (Population, Intervention, Comparison, Outcome) strategy was developed. As summarized in Supplementary Table S2, the PICO question addressed was “Does the supplementation with AAs in IMDs improve metabolic stability and biochemical parameters?”

The literature search was conducted on three databases, namely PubMed/Medline, Scopus, and Cochrane Library. The complete search strategy terms are listed in Supplementary Table S3. The inclusion criteria consisted of only original studies written in English, with no restrictions on age or year of publication. Scientific articles, including systematic reviews, consensus statements, guidelines, observational studies, case reports, and case series, were included in the review.

### 2.2. Identification of Relevant Studies

Study selection was performed by two authors independently (double-blinded). After the removal of duplicate records, all articles were screened according to their titles and abstracts. Articles were excluded for the following reasons, namely background articles or irrelevant, wrong dietary exposure, wrong population, wrong publication type, incorrect outcome, and foreign language. After, full text selection was performed, and in case of disagreement about the eligibility of any articles, the opinion of a third author was sought.

### 2.3. Study Selection and Evaluation

Selected articles were fully analyzed to extract information about *Does the supplementation with AAs in IMDs improve metabolic stability and biochemical parameters?* We identified the following main outcomes: biochemical outcomes (correction of secondary deficiencies, reduction in toxic metabolite plasma levels, improvement in neurotransmitter homeostasis, increased plasma levels of supplemented amino acid, reduction in toxic metabolite in brain concentrations) and clinical outcomes (improvement in muscle strength, improvement in respiratory function, improvement in vision, reduction in epithelial damage, improvement in neurological symptoms, improved neurodevelopment and growth, prevention of stroke-like episodes). Specific information is provided in Supplementary Table S2.

On the basis of the systematic research and evidence gathered, the recommendations for clinical practice were then formulated. Specifically, the quality of evidence was graded according to the Oxford Centre for Evidence-Based Medicine 2011 Levels of Evidence (LoE) [12], as detailed in Table 1.

## 3. Results

**Figure 1:**
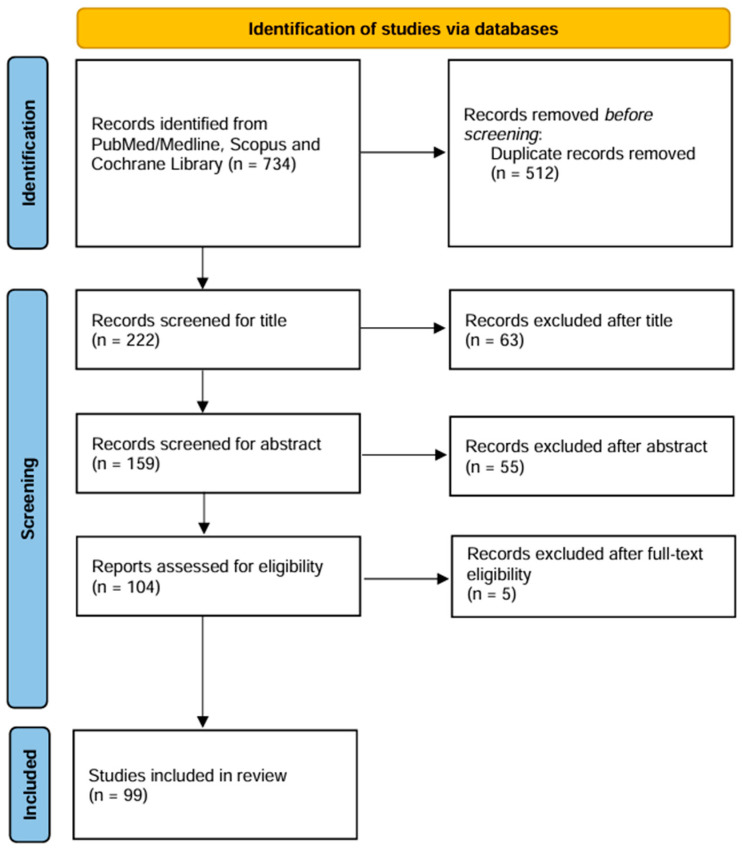
Flow diagram of the literature search process.

The initial systematic search retrieved a total of 734 articles published between 1976 and 2025. After removal of duplicates, a total of 222 articles were screened for title and abstract. The remaining 104 articles were screened for full text. Considering also the articles added manually by checking the reference list, we finally included 99 articles. Figure 1 presents the flowchart of the review process. The list of IMD included in the final review is presented in Supplementary Table S4, together with their main etiological and therapeutic characteristics.

### 3.1. Branched Chain Amino Acids (BCAAs): Leucine, Isoleucine, and Valine

*IMDs that could benefit from single AA supplementation:*

- *Urea cycle disorders (UCDs);*
- *Maple Syrup Urine Disease (MSUD);*
- *Methylmalonic (MMA) and Propionic (PA) acidemia;*
- *Glycogen storage disease due to acid maltase deficiency (Pompe Disease);*
- *t-RNA synthetase defects Isoleucyl-tRNA synthetase (IARS) and Leucyl-tRNA synthetase (LARS).*

The urea cycle has a crucial role in human metabolism since it is involved in ammonia detoxification through the process of ureagenesis. Urea cycle disorders (UCDs) are inborn errors due to defects in the six enzymes and two transporters required for the flux through the urea cycle [13]. To control hyperammonemia, patients affected by UCDs are treated with low-protein diets, essential amino acid supplementation (when stricter restriction of natural protein intake is necessary), combined with nitrogen scavengers in various formulations (sodium benzoate, sodium/glycerol phenylbutyrate), and supplementation of missing semi-essential amino acids (arginine, citrulline). Treatment with phenylbutyrate may decrease the circulating levels of BCAAs, especially those of Leu [13]. The 2013 European survey on UCDs management showed that the supplementation of BCAAs occurred only in 3% of patients with a highly variable dose up to 4 g/day protein equivalent [14], and the two most common conditions were ornithine transcarbamylase (OTC) deficiency and citrullinemia. Phenylbutyrate, which primarily reduces the levels of glutamine by producing phenylacetyl-glutamine, also impacts the metabolism of BCAAs. The depletion of the intracellular glutamine pool may increase the transamination of BCAAs by using their amino groups to produce glutamate, via the α-ketoglutarate pathway [15]. Phenylbutyrate could also, directly or indirectly, activate the branched chain α-keto acid dehydrogenase complex, resulting in increased BCAAs oxidation and reducing their concentration in plasma [15,16]. Careful monitoring of the plasma BCAAs profile in patients treated with sodium/glycerol phenylbutyrate therapy is therefore recommended to provide targeted supplementation of specific BCAAs and correct deficiencies [13]. Moreover, supplementation with BCAAs or other missing essential amino acids should always be evaluated in relation to excessive restriction of natural protein intake to allow a prompt correction of secondary (selective) deficiencies [13].

Maple syrup urine disease (MSUD) is a rare autosomal recessive disorder caused by deficient activity of the branched-chain α-ketoacid dehydrogenase enzyme complex. Accumulation of the three BCAAs (Leu, Val, Ile), allo-isoleucine, and their corresponding α-ketoacids results in severe brain dysfunction, neurological sequelae, or even death. The mainstay of treatment includes a strict dietary regimen low in natural protein, combined with MSUD-specific synthetic amino acid formulas free of the offending BCAAs. However, in MSUD patients, the need for Val and Ile supplementation arises from protein restriction aimed at reducing blood Leu levels, which can lead to insufficient dietary intake of Val and Ile, resulting in low plasma concentrations [10]. In individuals not affected by MSUD, the BCKD complex maintains strict stoichiometric ratios between the three BCAAs, with plasma concentration ratios between Leu and Ile and between Leu and Val remaining close to 2:1 in various physiological contexts, including overnight fasting, protein loading, and catabolic status [17]. The nutritional deficiency of Ile and Val can cause severe epithelial damage, affecting the skin, eyes, and gastrointestinal tract, with prompt improvement following single amino acid supplementation of the missing amino acids [18,19,20,21]. A study on 36 MSUD patients, evaluated in both acute and chronic dietary management, recommended a limited and controlled supplementation of Ile and Val [22]. The systematic review of van Vliet et al. [10] indicated the supplementation of Ile and Val to resolve dermatitis (level of evidence 4–5) and to lower blood Leu levels (level 5) during acute metabolic decompensation. Moreover, Ile and Val can compete with Leu for brain uptake [22] and, if deficient, could become limiting factors for protein synthesis, resulting in high blood Leu concentrations [23,24]. Adequate levels of Val and Ile are always necessary to support protein synthesis and facilitate the reduction in Leu concentrations. The standard intake of Ile and Val varies depending on patients’ age, plasma concentrations, and also the degree of BCKD deficiency/activity, ranging approximately from 10 to 30 mg/kg/day [25]. In the long-term maintenance, plasma Leu concentration should be maintained at 75–200 μmol/L below 5 years of age and at 75–300 μmol/L after 5 years of age [25]. During acute episodes of decompensation, it is advised to increase the amount of Ile and Val to 20–120 mg/kg/day to achieve plasma concentrations of 400–800 μmol/L for each [18]. In the long-term dietary management of MSUD, it is important to strictly monitor the plasma amino acid profile to maintain an adequate protein intake by combining natural protein foods with BCAA-free protein substitute and, when needed, single Ile and Val supplements, aiming to reach the RDA and the optimal Leu:Ile:Val ratio of 2:1:1. Increased supplementation of Val and Ile should always be considered during episodes of metabolic decompensation with hyperleucinemia, even if blood Leucine values fall within the normal range.

**Figure 2:**
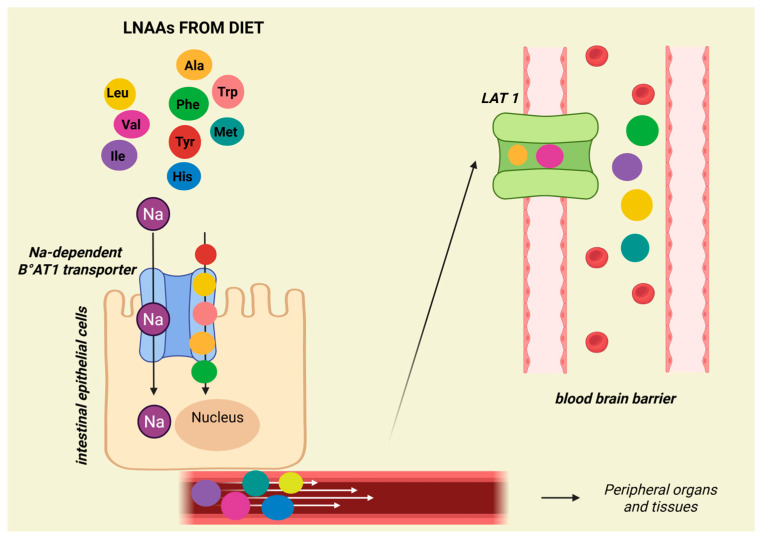
Mechanism of amino acid absorption across intestinal enterocytes and the blood–blood-brain barrier.

Propionic (PA) and Methylmalonic (MMA) acidemia, the most frequent organic acidemias, are characterized by the accumulation of propionic and/or methylmalonic acid due to deficiency of propionyl-CoA carboxylase (PCC) or methylmalonyl-CoA mutase (MUT). These two enzymes are involved in the catabolism of different precursors, which include the amino acids Val, Ile, threonine (Thr), and methionine (Met), odd-chain fatty acids, the cholesterol side chain, and propionic acid produced by the gut microbiota [26]. Treatment relies on the dietary restriction of natural proteins supplemented with a specific protein substitute and on chronic carnitine therapy [26]. The specifically designed protein substitutes for PA and MMA are free of the offending essential AAs but have a high Leu content [27]. An imbalance in Leu intake could adversely affect Ile and Val levels in plasma and CSF through a competitive mechanism, as the three BCAAs share common transporters for all large neutral AAs (leucine, valine, isoleucine, phenylalanine, tyrosine, histidine, methionine, tryptophan, and alanine) in intestinal epithelial cells (Na-dependent B°AT1) and at the blood–brain barrier (LAT1) (Figure 2) [28,29].

**Figure 3:**
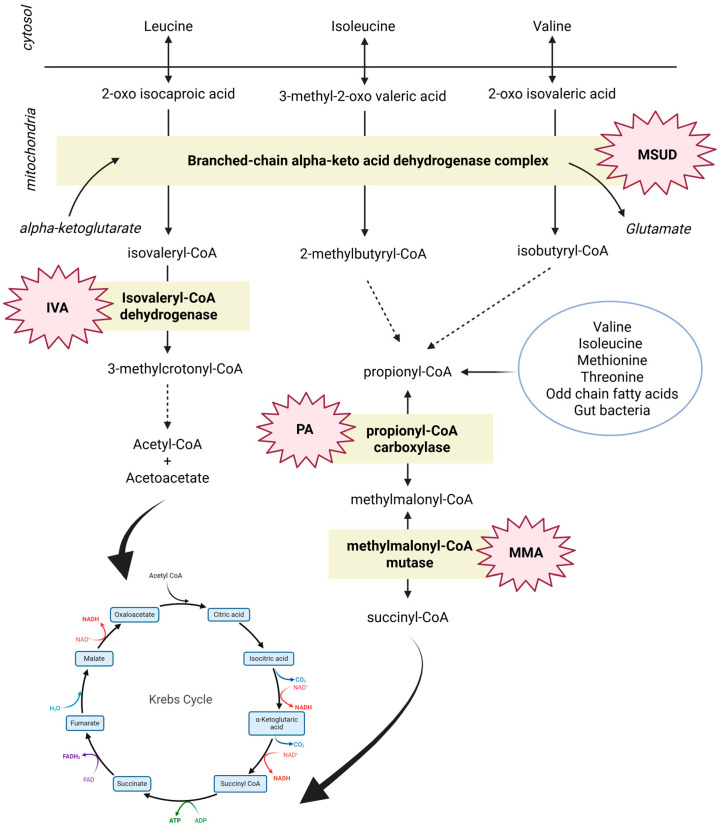
Illustration of the enzymatic steps involving BCAAs at the mitochondrial level. It depicts the enzymes that are deficient in MSUD, where the BCKAD complex is crucial for the catabolism of all BCAAs; in MMA and PA, where methylmalonyl-CoA mutase and propionyl-CoA carboxylase affect the metabolism of Ile and Val, as well as Met and Thr; and in IVA, where a deficiency in isovaleryl-CoA dehydrogenase impacts the metabolism of Leu.

Despite an adequate dietary intake of total protein, patients with MMA and PA may manifest low levels of BCAAs, suggesting that an excess of protein substitutes can lead to an imbalanced intake of Val and Ile compared to Leu, whose ingestion may exceed the WHO recommendations by four to five times, resulting in a iatrogenic reduction in essential AAs levels, poor growth, and an impact on cerebral AAs uptake/exchange [27]. Similar findings were reported in another study, which confirmed that the high amount of Leu in protein substitutes had a negative effect on plasma Val levels, leading to altered Leu/Ile and Leu/Val ratios [30]. The calculation of BCAA ratios could provide a better evaluation of whether to increase natural protein or to reduce protein substitutes. Moreover, another study [31] showed that patients with organic acidemias who ate only natural proteins tended to have a lower BMI. The study was based on the hypothesis that not only the amount of protein, and Leu in particular, but also its natural or synthetic origin must be considered, especially with regard to satiety. In patients from the Gugelmo study [31], plasma BCAAs levels were lower, especially in the supplemented patients: Leu levels, in particular, were low despite a quantitatively significant supplementation. The review of van Vliet et al. [10] reports that supplementation with Ile (48–340 mg/day) and Val (68–170 mg/day) has become, in some centres, a routine clinical practice to prevent EAAs deficiencies in MMA [32]. Similarly, the same review states that Ile supplementation (100 mg/day) is commonly used to prevent its deficiency, also in PA. For both diseases, combined supplementation with Val (70–170 mg/kg/day) and Ile (50–350 mg/kg/day) is suggested [10]. Recent GMDI guidelines for the nutritional management of PA recommend increasing natural protein rather than using Val and/or Ile supplementation to correct their plasma levels [33]. Considering Leu’s key role as a metabolic regulator, the combination of natural protein restriction with the use of protein substitutes that are excessively rich in Leu could result in an imbalance in BCAAs dietary content, with high Leu/Val and/or Leu/Ile ratios [34], highlighting the need to optimize the composition of medical foods for MMA and PA. It is therefore necessary to avoid excessive natural protein restriction and to carefully consider the nutritional importance of single-AA supplementation, especially in patients who have already reached their maximum protein tolerance. Figure 3 outlines the metabolic pathways involving the BCAAs, including MSUD, PA, and IVA, illustrating how sodium phenylbutyrate can impact BCAAs levels.

In Glycogen Storage Disease type II, also called Pompe Disease, the deficiency of the lysosomal enzyme acid α-glucosidase leads to a disease whose treatment is mainly based on enzyme replacement therapy (ERT) with recombinant human GAA (rhGAA). ERT is effective in improving or stabilizing the disease course in both infantile and late-onset forms [35]. The guidelines recommend monitoring the nutritional status and nutrient (protein) intake, following a diet composition with 25–30% protein, and ensuring an adequate caloric intake to avoid catabolism [35]. In adult patients, a high protein diet (1.2–1.4 g/kg protein per day) is recommended to counteract muscle protein depletion by supplying AAs for protein synthesis [35]. It has been reported that in adult Pompe patients, supplementation with a high intake of branched-chain AAs had a positive impact on respiratory function and muscle strength, nutritional status, and serum levels of branched-chain amino acids [36]. A case series [37] on five adult patients with Pompe Disease compared Leu production on a normal diet and after six months on an isocaloric high-protein diet (16–22% protein). After the high-protein diet, the leucine production rate decreased in four out of five patients.

Considering aminoacyl-tRNA synthetase (ARS) deficiencies, isoleucyl-RS (IARS), and leucyl-RS (LARS), the study of Kok et al. [38] set the following treatment scheme in two patients with ARS deficiencies: for IARS, Ile 35–70 mg/kg/day in three dosages, for LARS, Leu 35–100 mg/kg/day, and protein fortification of 2.5 g/kg/day. Beneficial effects in growth, infections, and oxygen dependency were shown. The same study highlighted an in vitro increase in sensitivity to ARS specific amino acid deprivation in patients’ fibroblasts, observing a mechanism of episodic insufficient aminoacylation. More studies are needed to demonstrate the power of AA supplementation in patients with ARS deficiencies. A recently published study described an infant with LARS1 deficiency and hypoalbuminemia who underwent a one month of Leu supplementation (300 mg/day) combined with a high-protein diet, with no observed effect on albumin levels and no reported side effects, and a report on a patient with IARS1 deficiency who received Ile supplementation (200 mg/kg/day), which led to improvements in feeding, development, and susceptibility to infections [39].

### 3.2. Aromatic Amino Acids (AAAs): Phenylalanine, Tryptophan, and Tyrosine

*IMDs that could benefit from single AA supplementation:*

- *Phenylketonuria (PKU);*
- *Phenylalanyl-tRNA synthetase (FARS).*

**Figure 4:**
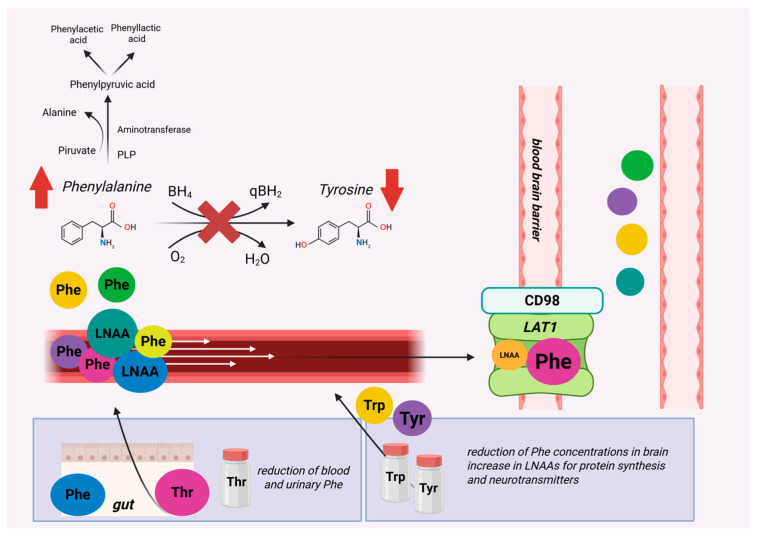
Illustration of single amino acid supplementation strategies in Phenylketonuria. It shows how supplementation with Tryptophan, Threonine, and Tyrosine can help compete with phenylalanine at the blood–brain barrier and reduce Phenylalanine levels.

Although the gold-standard of Phenylketonuria (PKU) therapy is dietary treatment, the use of large neutral amino acids (LNAAs) as a complementary or alternative therapy is gaining more and more importance because, through a competitive mechanism, LNAAs reduce the brain concentration of Phe by preventing its neurotoxic effect and also reduce exogenously based peripheral hyperphenylalaninemia. In PKU, LNAAs should not be given before 12 years of age and during pregnancy [40]. Among the LNAAs, Tyrosine (Tyr) and Tryptophan (Trp) hold a key role as they are essential for neurotransmitter synthesis, which could favour a normalization of neurotransmitter homeostasis in PKU. In patients with PKU, Tyr supplementation is indispensable due to its impaired endogenous synthesis from Phe, and it is added to all Phe-free L-amino acid protein substitutes, providing 9 to 11% of their L-amino acids. Guidelines indicate that the optimal amount of Tyr in a low-Phe diet remains undefined but that additional Tyr supplementation beyond the amount added in Phe-free protein substitutes does not confer any demonstrated clinical benefit. [40]. Moreover, a recent Cochrane review [41] highlighted that, on the basis of the currently available evidence, it is not possible to draw unequivocal conclusions about its efficacy. The principle behind LNAA supplementation relies on the shared transporter LAT1-CD98, which can limit Phe entry both peripherally and centrally. Despite conflicting results regarding the ability of this approach to reduce peripheral Phe levels, the study by Pietz et al. [42] shows a reduction in Phe influx in the brain thanks to Phe+LNAA supplementation. Studies by van Spronsen et al. [43,44,45] showed that brain protein synthesis is inversely proportional to the central concentration of Phe and that it is reduced at low Tyr concentrations. Supplementation with LNAAs can decrease brain concentrations of Phe and simultaneously supply the brain with LNAAs essential for protein synthesis. By ensuring an adequate supply of Tyr and Trp centrally, LNAAs would be able to increase neurotransmitter synthesis for dopamine and serotonin. The systematic review of van Vliet et al. [10] on single AA supplementation suggested threonine supplementation (50 mg/kg/day; approximately 60% of unsupplemented threonine intake) to decrease blood Phe based on observations in PKU patients [46]. Regarding Trp, a study in adults with classical PKU showed that a higher dose of supplementation (100 mg/kg/day of Trp) did not increase melatonin levels, but dopamine levels were increased with the higher dose of Tyr supplementation (200 mg/kg/day of Tyr). Serotonin synthesis appears to be suppressed by high Phe levels at the Trp hydroxylase level [47]. Figure 4 summarizes the possible single amino acid supplementation for Phenylketonuria.

In patients with Phenylalanyl-tRNA synthetase (FARS), a supplementation of 40–100 mg/kg/day Phe was used [38], showing, in a patient, beneficial effects in growth, head circumference, development, infection resistance, and oxygen dependency. Oswald et al. [48] recently described the case of a 3-year-old girl with mitochondrial FARS2 deficiency in whom Phe supplementation was used; the patient showed a clear improvement in all areas tested (motor function tests and quality-of-life questionnaires), particularly in motor skills and postural stability. More studies are needed to confirm these preliminary results on Phe supplementation in FARS.

### 3.3. Sulfur Amino Acids (S-AAs): Cysteine and Methionine

*IMDs that could benefit from single AA supplementation:*

- *Cystathionine β-synthase-deficient homocystinuria;*
- *Methylmalonic acidemia with homocystinuria, type cblC;*
- *Methylcobalamin deficiency type cblG;*
- *Methionyl-tRNA synthetase (MARS).*

Met is a sulfur-containing amino acid, introduced into the body through the diet, and plays a key role in several biochemical pathways. In classical homocystinuria (HCU) due to the deficient activity of cystathionine β-synthase (CBS), elevated levels of homocysteine (Hcy) are linked to clinical manifestations. Lifelong diet therapy includes the restriction of natural protein and the use of Met-free essential AAs mixtures, combined with betaine and vitamin B6, to reduce Met intake and lower Hcy levels [49]. Moreover, cysteine, which is a conditionally (semi) essential AA in CBS deficiency, is added to protein substitutes (around 30–50 mg of cysteine per g of protein equivalent) [10] or supplemented as single AA in patients with severe deficiency, not only to correct its deficiency but also to reduce blood Hcy levels. However, no study has explored the potential homocysteine-lowering effect of cysteine supplementation in CBS-deficient patients. The systematic review of van Vilet et al. [10] reports possible mechanisms. Cysteine may shift homocysteine from its protein-bound form to a low-molecular-weight form, aiding urinary clearance; reduce homocysteine formation from methionine; and promote homocysteine remethylation to methionine. Studies in rats support the first hypothesis, showing that cysteine decreases protein-bound homocysteine and total levels when on a low-protein/methionine diet. A fourth theory suggests that supplementing cysteine (when levels are <170 μmol/L) could lower free plasma Hcy to maintain a stable thiol redox balance [50].

Remethylation disorders comprise “isolated” conditions affecting the conversion of Hcy to Met (cblD-HC, cblE, cblG, and MTHFR) or disorders of intracellular processing of vitamin B12 (cblC, cblD-HCY/MMA, cblF, cblJ, cblX), causing a combined deficiency in the synthesis of methyl- and adenosyl-cobalamin, the cofactors for methylmalonyl-CoA mutase and methionine synthase [51]. In isolated remethylation disorders, plasma total Hcy is elevated and Met is reduced, while combined defects are associated with increased Hcy, low to normal Met, and increased methylmalonic acid (MMA).

Treatment of the cblC defect, the most frequent remethylation disorder, consists of parenteral hydroxycobalamin administration, combined with oral betaine, folate, and carnitine [51]. More rarely, Met supplementation has been utilized to improve the metabolic profile. Current guidelines recommend maintaining Met plasma levels in the normal range and, if necessary, this may be achieved by oral Met supplementation [51]. A recent systematic review on late-onset methylmalonic acidemia and homocysteinemia (cblC disease) [52] found that methionine levels were decreased in 49% of the total measurements. However, to date, there is no evidence on the effect of Met supplementation in cblC patients. A 2014 case-report [53] on three patients with remethylation defects (one with methionine synthase deficiency (cblG) and two with methyltetrahydrofolate reductase deficiency (MTHFR), all intubated for respiratory failure, described clinical improvement thanks to medical treatment with betaine and folinic acid supplementation in all three, methionine in two, and cobalamin supplementation in two. Another recent study on three patients diagnosed with cblG [54] reported that Met supplementation (10–12 mg/kg/day), together with hydroxocobalamin, betaine, and folinic acid, can help reduce homocysteine levels and increase methionine.

With reference to ARS deficiencies, in methionyl-tRNA synthetase (MARS) deficiency, protein intake with methionine supplementation from 86 to 150 mg/kg/day led to improvements in growth, neurodevelopment, and pulmonary function in two affected patients [55]. Delacourt et al. [56] reported that, in children with pulmonary alveolar proteinosis (PAP), Met supplementation (starting at 80 mg/kg/day and increased until reaching levels between 45 and 500 µM) was associated with respiratory improvements. A 2024 study [57] showed a positive outcome in three cases of lung transplantation for PAP, who were under methionine supplementation (62 mg/kg/day), whereas another case without supplementation had fatal PAP recurrence. A 2025 study [39] summarizes several studies in which supplementation ranged from 50 to 150 mg/kg/day, depending on the specific cases, with some protocols administering divided doses every 6 h throughout the day.

### 3.4. Urea Cycle Amino Acids (UCD-AAs): Arginine, Citrulline, and Ornithine

*IMDs that could benefit from single AA supplementation:*

- *Urea Cycle Disorders (UCDs);*
- *Citrin deficiency;*
- *Lysinuric protein intolerance (LPI);*
- *Glutaric Acidemia type 1;*
- *Pyridoxine-dependent epilepsy (PDE) and antiquitin deficiency (ATQ);*
- *X-linked creatine transporter deficiency (CRTR-D);*
- *Guanidinoacetate methyltransferase deficiency (GAMT);*
- *ALDH18A1-related De Barsy syndrome (P5CS deficiency);*
- *Mitochondrial encephalomyopathy, lactic acidosis, and stroke-like episodes syndrome (MELAS).*

In the majority of urea cycle disorders, arginine (Arg) and/or citrulline (Cit) become conditionally (semi-) essential AAs due to impaired synthesis, and their supplementation is necessary not only to correct deficiencies but also to provide substrate(s) for ureagenesis [13]. In this regard, Arg and/or Cit supplementation aims at maximizing the excretion of ammonia during acute decompensation and improving metabolic stability in the long-term management [13]. With the exception of Argininemia, Arg can be supplied to all patients with UCDs, while Cit should not be supplemented in Argininosuccinate Synthase 1 (ASS1) and Argininosuccinate Lyase (ASL) deficiencies [13]. A recent longitudinal study on 79 patients with UCDs caused by OTC, Carbamoylphosphate synthetase I (CPS1), N-acetyl glutamate synthetase (NAGS), and N-acetyl glutamate synthetase (ORNT1) defects highlighted the importance of Cit supplementation, either alone or in combination with Arg (Cit mean dose 150 mg/kg/d, max dose 400 mg/kg/day; Arg mean dose 200 mg/kg/day, max dose 300 mg/kg/day) [58]. Treatment with Cit as a monotherapy increased plasma Arg, with values significantly higher than those observed with Arg therapy alone or with combined Arg and Cit [58]. Ornithine (Orn) supplementation has been used with contradictory results in some patients with Hyperornithinemia–Hyperammonemia–Homocitrullinuria (HHH) syndrome in an attempt to correct Orn depletion in the mitochondria; however, its use is no longer recommended [59].

Citrin deficiency (CD), a recessive disease caused by mutations in the SLC25A13 gene, is characterized by three different age-dependent phenotypes: (a) neonatal intrahepatic cholestasis; (b) failure to thrive and dyslipidemia in children; and (c) citrullinemia type 2, with hyperammonemic attacks in adolescent or adult patients. Unlike other UCDs, in CD, a dietary regimen rich in protein, fats, and medium-chain triglycerides (MCT) and low in carbohydrates is recommended [60,61]. Arg supplementation in a CD patient was effective in lowering and maintaining low ammonia levels in blood [62]. However, plasma Arg levels are often elevated in CD, raising questions about Arg supplementation [60].

Lysinuric protein intolerance (LPI) is a rare autosomal recessive disorder of AA transport caused by mutations in the *SLC7A7* gene, coding for the y+L amino acid transporter 1 (y+LAT1), which transfers cationic AAs Orn, Arg, and Lys from the cell to the extracellular space. The transporter y+LAT1 is expressed in enterocytes, renal tubular cells, and monocytes/macrophages and its deficiency causes a complex phenotype that includes intermittent hyperammonemia with intolerance to protein-rich food (due to a functional urea cycle disorder), failure to thrive, renal Fanconi syndrome, and severe/life-threatening symptoms that include pulmonary alveolar proteinosis and hemophagocytic lymphohistiocytosis [63]. Treatment of LPI aims to prevent hyperammonemia by reducing natural protein intake combined with Cit supplementation (100 mg/kg/day in four divided doses) to increase missing urea cycle intermediates [63,64]. The review of van Vliet et al. [10] also recommends long-term citrulline supplementation (0.5–1.1 mmol/kg/day) to restore proper urea cycle function, while its effect on bone mineral density requires more evidence [65,66,67,68,69].

The management of Glutaric Acidemia type 1 (GA1) is based on natural protein restriction to limit lysine (Lys) and tryptophan intake, together with Lys-free, low-Trp, and Arg-supplemented protein substitutes and carnitine administration [70]. Arginine is an essential AA that competes with lysine for the transporter SLC7A1 at the blood–brain barrier (BBB), reducing the downstream metabolic products. Recommendations for Arg supplementation have been discussed in the work of Van Vliet et al. [10], for both routine and acute management, considering 100–150 mg/kg/day (but with dietary lysine/arginine intake ratio of 0.5–0.8 for routine and higher for acute episodes, aiming to reach a dietary lysine/arginine intake of 0.15–0.20 (mg:mg) [10].

Along with GA1, studies have also highlighted the importance of arginine supplementation in lysine-restricted diets for patients with neurometabolic disorders such as pyridoxine-dependent epilepsy (PDE) [71], considering the competition at the BBB of these two AAs [72]. Evidence is limited to observational studies on patients with PDE [73,74,75], who were already on a lysine-restricted diet and pyridoxine treatment, which further reduced toxic metabolites and, in some cases, improved neurodevelopmental outcomes. This approach is called triple therapy and consists of dietary lysine restriction, pyridoxine therapy, and arginine supplementation.

Guanidinoacetate methyltransferase deficiency (GAMT) is an inherited disorder of creatine synthesis. The restriction of Arg intake, combined with the supplementation of Orn (100–400 mg/kg/day), was proposed to decrease arginine and guanidinoacetate (GAA) levels in the blood [10]. Higher Orn supplementation (600–800 mg/kg/day) may improve the GAA-lowering effect of dietary arginine restriction [10].

Arginine, together with glycine and creatine, has been used for the improvement in symptoms in X-linked creatine transporter deficiency (CRTR-D). In a case of a female with a de novo pathogenic SLC6A8 variant with active symptoms [76], the creatine, arginine, and glycine supplementation improved symptoms and creatine concentrations at magnetic resonance spectroscopy. Another recent case report [77] on a 16 year-old female who had been receiving supplementation since 14 years of age a supplementation with creatine (400 mg/kg/day), arginine (400 mg/kg/day), and glycine (150 mg/kg/day, increased at 180 mg/kg/die), together with pharmacological treatment with Brivaracetam (200 mg/day) and Eslicarbazepine (800 mg/day), showing amelioration of symptoms and creatine levels in brain. The same work reported the results of a review on 33 female cases, highlighting that supplementation should be tested in heterozygous female patients with CTD, while in male patients, the same supplementation yielded unclear results [77]. A 2024 review [78] on supplementation in patients with CTD found that arginine and glycine combination could be a promising therapy, while arginine alone remains controversial.

In ALDH18A1-related De Barsy syndrome (pyrroline-5-carboxylate synthetase deficiency, P5CSD), low levels of proline (Pro), Orn, Arg, and Cit may be found, together with mild fasting hyperammonemia. In a single case report, Arg supplementation (150 mg/kg/day) ameliorated fasting plasma ammonia, Pro, and Orn levels, normalized brain creatine concentration (as detected by MRS) and positively impacted psychomotor development, revealing that in P5CSD, Arg becomes a conditionally (semi-) essential AA [79].

In mitochondrial encephalomyopathy, lactic acidosis, and stroke-like episodes syndrome (MELAS), Arg supplementation, as a precursor of nitric oxide (NO), is used for acute attacks, based on the hypothesis that Arg supply could increase the synthesis of NO, promoting a vasoactive effect on brain perfusion and restoring a physiological state after stroke-like episodes [80,81]. However, the exact mechanisms underlying clinical improvements are still not well defined [81]. A recent systematic literature review reported the effectiveness of IV Arg in improving symptoms during acute attacks of MELAS and of oral Arg supplementation in the prevention of further stroke-like episodes [82]. Conversely, the conclusions of another systematic review highlighted that both IV and oral Arg have no demonstrable clinical benefit in either the acute or prophylactic treatment of MELAS [83]. These contradictory conclusions highlight the need for further studies aimed at better understanding the mechanisms of action and assessing the impact of Arg therapy in treating and preventing stroke-like episodes in MELAS [80,81,82,83].

### 3.5. Other Essential Amino Acids (EAAs): Threonine and Lysine

*IMDs that could benefit from single AA supplementation:*

- *Ornithine aminotransferase deficiency (OAT);*
- *Phenylketonuria (PKU);*
- *Lysinuric protein intolerance.*

In patients with ornithine aminotransferase deficiency (OAT), Lys supplementation (10–15 g/day) decreased plasma Orn levels by increasing its urinary excretion [84,85], considering that Orn, Arg, and Lys share the same renal transport system [10]. A recent expert consensus included, among the nutritional interventions for OAT deficiency, the lifelong restriction of dietary protein, essential amino acids, and creatine supplements but not Lys supplementation [86].

Threonine (Thr) is an essential large neutral AA, whose blood concentration in patients with PKU was found to be inversely related to blood Phe levels [10,87] and nonlinearly correlated with Phe levels in patients with hyperphenylalaninemia [88]. In patients with PKU, single threonine (Thr) supplementation (50 mg/kg/day) has been shown to reduce both blood and urinary Phe because of competition at the gut–blood barrier [46,89]. Figure 4 summarizes the possible single amino acid supplementation for Phenylketonuria.

Blood Lys levels are usually found below normal in patients with Lysinuric Protein Intolerance [69], and its deficiency could contribute to some of the clinical manifestations, in particular to abnormal collagen maturation and osteopenia due to low hydroxylysine synthesis from Lys [63]. Lys supplementation (20–30 mg/kg/day, in 3–4 doses per day) is therefore recommended to correct its deficiency, even in the absence of an overt effect on osteopenia [63].

### 3.6. Other Non-Essential Amino Acids (Non-EAAs): Alanine, Glycine, Glutamine, Proline, and Serine

*IMDs that could benefit from single AA supplementation:*

- *Isovaleric acidemia (IVA);*
- *X-linked creatine transporter deficiency (CRTR-D);*
- *Glutamine synthetase deficiency;*
- *Ornithine aminotransferase deficiency (OAT);*
- *ALDH18A1-related De Barsy syndrome (P5CS deficit);*
- *Seryl-tRNA synthetase 1 (SARS);*
- *Neurometabolic disorder due to serine deficiency;*
- *3-Phosphoglycerate dehydrogenase deficiency (3-PGDH deficiency);*
- *Deficiency of phosphoserine aminotransferase (PSAT);*
- *GRIN-related disorders;*
- *Glycogen storage disease due to acid maltase deficiency (Pompe Disease).*

Isovaleric acidemia is a disorder of Leu catabolism due to deficient activity of isovaleryl-CoA dehydrogenase [90]. The clinical manifestations are similar to those seen in other organic aciduria (i.e., PA and MMA), with a generally less severe disease course [90]. Treatment is based on a protein-restricted diet supplemented with Leu-free AA mixtures [90]. By exploiting the possibility of forming isovaleryl-CaA esters through the action of carnitine acyl transferase and glycine-N acylase, the combined treatment with carnitine and Gly supplementation is recommended to buffer the intramitochondrial excess of isovaleryl-Coa [91]. Treatment with Gly supplements (up to 300 mg/kg/day) allowed for a reduction in circulating levels of isovaleric acid by increasing the urinary excretion of N-isovalerylglycine during acute metabolic decompensation events [92,93,94]. The review by van Vliet et al. stated that glycine supplementation is effective both for the acute and chronic management of IVA [10], as also confirmed by Mutze et al. [90], who recommend glycine supplementation (150 mg/kg/day up to 300 mg/kg/day) for pharmacologic detoxification, together with carnitine supplementation. Unlike patients with classical IVA, individuals with mild IVA variants identified by newborn screening present excellent clinical outcomes, regardless of metabolic maintenance therapy [95].

Gly, together with creatine and Arg, is also indicated for X-linked creatine transporter deficiency (CRTR-D). *See the urea cycle amino acids paragraph.*

Glutamine is the most abundant free amino acid present in the human body, playing important roles in different metabolic pathways; moreover, it is a precursor for neurotransmitters. Blood levels of this AA can be decreased in some patients affected by Glutamine synthetase deficiency, an ultra-rare disease whose treatment depends on the symptoms [96]. One study [97] on a patient with this disorder showed improvement in brain function thanks to enteral glutamine supplementation for four weeks, starting with a dose of 17 mg/kg/day and increasing progressively to 1020 mg/kg/day.

In OAT, proline supplementation may be employed to restore a deficiency of this AA [10], with a reported positive effect on vision in some patients [98].

The use of Pro for ALDH18A1-related De Barsy syndrome is discussed in the *urea cycle amino acids paragraph*, together with creatine and Arg, Orn, and Cit.

**Figure 5:**
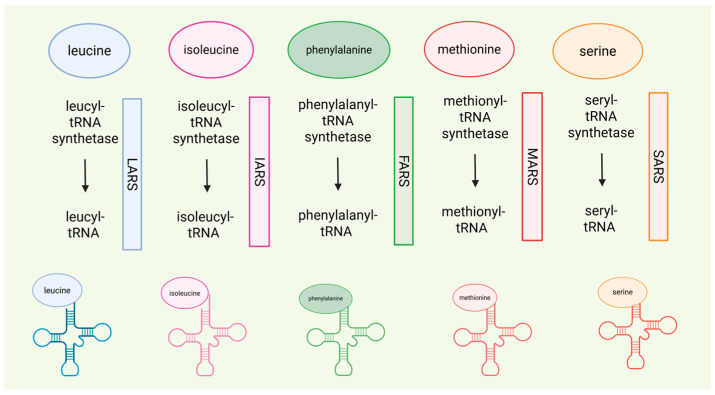
Schematic overview showing single amino acid supplementation strategies in ARS deficiencies. It depicts how supplementation with specific amino acids—serine (SARS), phenylalanine (FARS), methionine (MARS), leucine (LARS), and isoleucine (IARS)—can help restore metabolic balance and support proper protein synthesis in the context of defective aminoacyl-tRNA synthetases.

With reference to aminoacyl-tRNA synthetase, a study [38] on ARS deficiencies showed that serine supplementation ranging from 85.7 to 97.5 mg/kg/day in fibroblasts of patients with Seryl-tRNA synthetase 1 (SARS) enabled improvements in height and development, as well as resolution of microcephaly. Figure 5 summarizes the ARS deficiencies discussed in this review.

In neurometabolic disorders due to serine (Ser) deficiency, Ser supplementation is part of the early treatment, and high doses of Ser are necessary to correct its deficiency and restore normal values in plasma and cerebrospinal fluid [99]. Young individuals may benefit from oral Ser therapy, starting with 200–400 mg/kg/day divided into 4–6 doses and reaching 500–700 mg/kg/day to prevent recurrence of seizures [99]. Combined with Ser therapy, Gly supplementation can also be considered. In 3-Phosphoglycerate dehydrogenase deficiency (3-PGDH deficiency), Gly supplementation (200–300 mg/kg/day) together with serine has shown improvements in seizure in a case report [100]. Gly (200 mg/kg/day) and Ser supplementation in the deficiency of phosphoserine aminotransferase (PSAT) allowed for better seizure control [101].

GRIN-related neurodevelopmental disorders (GRIN-NDD) comprise a phenotypic spectrum caused by pathogenic variants in one of the four GRIN genes (*GRIN1*, *GRIN2A*, *GRIN2B*, and *GRIN2D*), all of which encode different subunits of the N-methyl-D-aspartate receptor (NMDAR) for glutamate [102]. Ser mediates a co-agonist effect on NMDA receptors in neurons, via its enantiomer D-serine, which is generated from L-serine by serine racemase [103], exhibiting a significant modulatory effect on NMDA receptor-mediated neurotransmission and synaptic plasticity. Numerous preclinical and clinical studies suggest that Ser supplementation may be effective in reducing cognitive dysfunction in patients with schizophrenia or major depressive disorder [104]. Ser supplementation in a 5-year-old patient with GRIN2B-related developmental delay, intellectual disability, and autism spectrum disorder resulted in significant improvements in all neurodevelopmental assessments, suggesting that Ser supplementation could mitigate the neurological manifestations related to NMDA receptor dysfunction [105]. A recent open-label non-randomised single-arm study conducted to analyse the response to Ser in children with loss-of-function variants in GRIN genes enrolled 24 patients and confirmed significant improvements in behaviour, motor, and quality of life, with a more pronounced response in patients with milder phenotypes [106]. Ser was administered orally at 250 mg/kg/day, divided into three doses for two weeks, and increased to 500 mg/kg/day in the third week, not exceeding 30 g/day for patients weighing ≥60 kg.

In Pompe disease, guidelines report that alanine (Ala) supplementation has been proposed to reduce muscle protein turnover and to potentially improve muscle function [35]. Oral supplementation with Ala, a gluconeogenic AA capable of decreasing BCAA catabolism, of promoting glucose storage, and of reducing leucine oxidation [107] was administered to a 65 year-old woman with Pompe disease (powder mixed into a drink at a dose of 0.14 g/kg per day in three divided doses) for three months, resulting in a 15% increase in total body protein, with no improvements in muscle function, while the patient reported feeling worse after treatment [108]. Ala supplementation in five late-onset Pompe disease patients reduced protein turnover and catabolism, decreased resting energy expenditure, and lowered leucine flux and oxidation compared to seven healthy controls [109]. Before the advent of ERT, a late-infantile Pompe disease patient with left ventricular hypertrophy and respiratory insufficiency requiring mechanical ventilation was treated from the age of 2.5 years with Ala supplementation (2 g/kg/day), a dose representing a 50-fold increase compared with the intake of a standard diet. After three years, cardiomyopathy improved with only mild septum hypertrophy, while the severe generalized myopathy persisted; plasma Ala levels were increased, and no side effects were recorded [110]. A recent article on a 9-year-old child with late-infantile Pompe disease on ERT reported that Ala supplementation, up to 0.6 g/kg/day (for a total of 18 g/day), resulted after nine months in increased body fat mass and decreased resting energy expenditure [111].

## 4. Conclusions

In conclusion, this study represents the first comprehensive analysis of the potential benefits of single AA supplementation across the broad spectrum of inherited and rare metabolic diseases. Table 2 presents single AA supplementation with available specific doses, according to the levels of evidence [12]. Although this work represents a preliminary step in addressing this complex issue, it is important to acknowledge that the current scientific evidence is of limited quality. With few exceptions, most studies rely on individual patient case reports, which are insufficient to draw broad conclusions. The rarity of these disorders highlights the need for multicenter observational or interventional studies to collect and analyze widely applicable data. Furthermore, several critical parameters remain inadequately defined. Future research must establish the plasma amino acid levels that indicate the need for supplementation, define appropriate dosages (in g/day or mg/kg/day), determine the optimal treatment duration, and, crucially, identify the target plasma ranges to be maintained. It is also essential to differentiate between supplementation protocols for long-term management in metabolically stable conditions and those required during acute metabolic events. Furthermore, specific recommendations on the appropriate age for supplementation are needed. Addressing these gaps will be crucial for refining therapeutic strategies and ensuring that AA supplementation reaches its full potential in the management of rare metabolic diseases.

## Figures and Tables

**Table 1:** Levels of evidence according to the Oxford Centre for Evidence-Based Medicine [12].

Level of Evidence	Type of Study
Level 1: High-Quality Evidence	1a: Systematic Review (with homogeneity) of Randomized Controlled Trials (RCTs) 1b: Individual Randomized Controlled Trial (RCT) with a Narrow Confidence Interval 1c: “All or None” Study
Level 2: Individual RCTs or cohort studies with dramatic effects	2a: Systematic Review (with homogeneity) of Cohort Studies 2b: Individual Cohort Study or Low-Quality RCT 2c: “Outcomes” Research or Ecological Studies
Level 3: Non-randomized controlled cohort/follow-up study	3a: Systematic Review (with homogeneity) of Case-Control Studies 3b: Individual Case-Control Study
Level 4: Case Series or Poor-Quality Cohort and Case-Control Studies	
Level 5: Expert opinion or reasoning without supporting evidence from clinical studies	

**Table 2:** Single AA supplementation in IMDs, with a specific dose (D) of mg/kg/day when available, according to the level of evidence (LoE) cited in Table 1. An X is used where there is no evidence for supplementation.

	*IMDs*										
*AAs*	MSUD	PA	MMA	IVA	UCDs (Not Arg1)	IARS	LARS	SARS	FARS	MARS	POMPE
***Leu***					LoE: 1a		LoE: 4 D: 35–100 mg/kg/day				X
***Ile***	LoE: 4 D: 10–30 mg/kg/day (routine) D: 20–120 mg/kg/day (acute episodes)	LoE: 4 D: 100 mg/day or 50–350 mg/kg/die	LoE: 4 D: 48–340 mg/day or 50–350 mg/kg/die		LoE: 1a	LoE: 4 D: 35–70 or 200 mg/kg/day					X
***Val***	LoE: 4 D: 10–30 mg/kg/day (routine) D: 20–120 mg/kg/day (acute episodes)	LoE: 4 D: 70–170 mg/kg/day	LoE: 4 D: 70–170 mg/kg/day		LoE: 1a						X
***Ala***											LoE: 4 up to 600 mg/kg/day on ERT
***Gly***				LoE: 4 D: 150–300 mg/kg/day							
***Arg***					LoE: 2b D: 200–300 mg/kg/day						
***Cit***					LoE: 2b D: 150–400 mg/kg/day						
***Met***										LoE: 4 D: 50–150 mg/kg/day	
***Phe***									LoE: 4 D: 40–100 mg/kg/day		
***Ser***								LoE: 4 85.7–97.5 mg/kg/day			
	***IMDs***										
***AAs***	**PKU**	**HCU**	**CblC**	**CblG**	**GA1**	**GAMT**	**CRTR-D**	**CD**	**LPI**		
***Gly***							LoE: 4 D: 150–180 mg/kg/day				
***Arg***					LoE: 4 D: 100–150 mg/kg/day		LoE: 4 D: 400 mg/kg/day	X			
***Orn***						LoE: 4 D: 100–800 mg/kg/day					
***Cit***									LoE: 4 D: 0.5–1.1 mmol/kg/day (long term)		
***Lys***									LoE: 4 D: 20–30 mg/kg/day		
***Cys***		X									
***Met***			X	LoE: 4 D: 10–12 mg/kg/day							
***Phe***											
***Tyr***	X										
***Thr***	LoE: 2b D: 50 mg/kg/day										
***Trp***	LoE: 2b D: 100 mg/kg/day										
	***IMDs***										
***AAs***	***P5CSD***	***MELAS***	***PDE***	***OAT***	***GS def.***	***Serine def.***	***3-PGDH***	***PSAT***	***GRIN 2B***		
***Gly***							LoE: 4 D: 200–300 mg/kg/day	LoE: 4 D: 200 mg/kg/day			
***Arg***	LoE: 4 D: 150 mg/kg/day	X	LoE: 3b								
***Orn***	X										
***Cit***	X	X									
***Lys***				LoE: 4 D: 10–15 g/day							
***Gln***					LoE: 4 From 17 mg/kg/day to 1020 mg/kg/day (enteral and parenteral)						
***Pro***	X			4							
***Ser***						LoE: 4 D: From 200–400 to 500–700 mg/kg/day			LoE: 2b Treatment: 250–500 mg/kg/day (without exceeding 30 g/day for patients weighing ≥60 kg)		

Abbreviations. Leu: Leucine: Ile: Isoleucine; Val: Valine; Gly: Glycine; Arg: Arginine; Orn: Ornithine; Cit: Citrulline; Lys: Lysine; Gln: Glutamine; Pro: Proline; Cys: Cysteine; Met: Methionine; Phe: Phenylalanine; Tyr: Tyrosine; Thr: Threonine; Trp: Tryptophan; Ser: Serine; Ala: Alanine; IMDs: Inherited metabolic disorders; MSUD: Maple Syrup Urine Disease; PA: Propionic Acidemia; MMA: Methylmalonic acidemia; IVA: Isovaleric acidemia; UCDs: Urea Cycle Disorders; IARS: Isoleucyl-tRNA synthetase; LARS: Leucyl-tRNA synthetase; FARS: Phenylalanyl-tRNA synthetase; SARS: Seryl-tRNA synthetase; MARS: Methionyl-tRNA synthetase; POMPE: Glycogen storage disease due to acid maltase deficiency; PKU: Phenylketonuria; CBS: Cystathionine β-synthase-deficient homocystinuria; CblC: Methylmalonic acidemia with homocystinuria; CblG: Methylcobalamin deficiency; GA1: Glutaric Acidemia type 1; GAMT: Guanidinoacetate methyltransferase deficiency; CRTR-D: X-linked creatine transporter deficiency; CD: Citrin Deficiency; LPI: Lysinuric Protein Intolerance; P5CSD: ALDH18A1-related De Barsy syndrome; MELAS: Mitochondrial encephalomyopathy, lactic acidosis, and stroke-like episodes syndrome; PDE/ATQ: Pyridoxine-Dependent Epilepsy/Antiquitin Deficiency; OAT: Ornithine aminotransferase deficiency; GS deficiency: Glutamine Synthetase deficiency; Serine deficiency: Neurometabolic Disorder due to Serine Deficiency; 3-PGDH deficiency: 3-Phosphoglycerate dehydrogenase deficiency; GRIN: GRIN-related neurodevelopmental disorders (GRIN-NDD); PSAT: deficiency of phosphoserine aminotransferase.

## Data Availability

No new data were created or analyzed in this study. Data sharing is not applicable to this article.

## Supplementary Materials

The following supporting information can be downloaded at https://www.mdpi.com/article/10.3390/genes16050502/s1: Supplementary Material S1: Role and metabolic impact of amino acids, Supplementary Figure S1: Branched-chain amino acid intake and oxidation lead to a nutritional-metabolic impact on fatty acid metabolism and fat mass deposition. Whey proteins, which contain BCAAs, including Leu, have a greater impact on insulin production, whereas casein influences growth hormone (GH) production. Both contribute to IGF-1 release. When the body’s capacity to utilize amino acids is exceeded, they undergo oxidation via BCKD, leading to the formation of short-chain acylcarnitines. Specifically, C5 further inhibits fatty acid beta-oxidation, promoting adipose tissue accumulation, Supplementary Table S1: Protein digestibility according to food sources, Supplementary Table S2: PICOS criteria for inclusion of studies, Supplementary Table S3: Research strategies employed on PubMed/Medline, Scopus and Cochrane library, Supplementary Table S4: A summary table of the main aetiological and therapeutic characteristics of each IMD is included in this review.
