# Supplementary material for: Single Amino Acid Supplementation in Inherited Metabolic Disorders: An Evidence-Based Review of Interventions
Source: Genes (Basel). 2025 Apr 27;16(5):502. doi: 10.3390/genes16050502 (PMC12111424; doi:10.3390/genes16050502)
Supplement: Supplementary file 1 [file genes-16-00502-s001.zip › genes-3563880-supplementary.pdf]

Supplementary Material

Supplementary Material S1. Role and metabolic impact of amino acids.

Amino acids are absorbed in the intestine via various mechanisms, including facilitated diffusion, active transport, and secondary active transport. Each mechanism employs specific transporters to facilitate amino acid movement across the intestinal membrane. Regarding AAs absorption and transport, ingestion of hydrolyzed AAs formulas leads to an increase in blood urea levels. AAs from hydrolyzed proteins enter the bloodstream more rapidly than those from whole proteins and may even be absorbed faster than free amino acids [1]. When using AA-based formulas (AAF), it is essential to balance AA intake to prevent excessive nitrogen excretion while ensuring adequate energy intake to promote protein anabolism. Thus, a ratio of 3–4.5 g protein equivalent per 100 kcal, corresponding to 12%–18% of total energy, has been suggested [1]. In paediatric clinical nutrition, single AAs are used in various conditions, including artificial nutrition (both enteral and parenteral), kidney failure, burns, malnutrition, and sarcopenia. Nowadays, the use of AAs supplements in sports is also becoming increasingly widespread. Protein intake levels are defined by the Dietary Reference Values (DRV) established by the European Food Safety Authority (EFSA) [2].

The three branched-chain amino acids (BCAAs), leucine (Leu), isoleucine (Ile), and valine (Val) are essential amino acids involved in key metabolic processes, including skeletal muscle metabolism, the activity of branched-chain keto acid dehydrogenase (BCKD), and the amination of branched-chain keto acids (BCKAs) to BCAAs [3]. Leu is exclusively ketogenic, while Ile is both glucogenic and ketogenic, and Val is solely glucogenic [4]. Plasma BCAA concentrations rise during prolonged starvation and in the postprandial state following a protein-rich meal [5]. BCAAs supplementation is a widely used strategy to promote anabolic pathways by activating the mammalian target of rapamycin complex 1 (mTORC1) signaling pathway, both in health (e.g., physical activity and sports) and in muscle-wasting diseases [6]. However, recent studies have shown that elevated circulating BCAAs levels are associated with a higher risk of cardiometabolic diseases, including obesity, diabetes, and cardiovascular diseases [7]. Despite the evidence, it remains unclear whether the relationship between high plasma BCAAs levels, dietary BCAA intake, and total protein intake reflects a metabolic dysfunction [8]. High plasma BCAA levels may result from alterations in BCAA-catabolizing enzymes, such as BCKD, which plays a key role in BCAA oxidation. This enzymatic alteration can lead to mTORC1 hyperactivation, which has been linked to insulin resistance [9,10]. The European Childhood Obesity Project Trial (CHOP), a multicenter, randomized, double-blind trial [11], assigned healthy term-born infants to receive either a starting formula and a follow-on formula with a higher (HP) or lower (LP) protein content. At two years of age, in the high-protein formula group, BCAAs and their breakdown products (the C3, C4, C5, C5-OH and C5:1 acylcarnitines) were significantly elevated, as were the essential AAs phenylalanine and methionine. In contrast, the acylcarnitines C5-DC, C6:1, C8:1, C12, as well as the non-essential AA glutamine, were higher in the low-protein formula group. The most abundant AAs in the plasma of the children fed with the HP formula included BCAAs, aromatic AAs (AAAs; phenylalanine, tyrosine, and tryptophan), methionine, lysine and proline. As plasma BCAA concentrations increased, C4 and C5 acylcarnitine levels rose until a breakpoint was reached. Indeed, BCAAs are transaminated by the branched-chain amino transferase into keto acids, which are then degraded by BCKD into short-chain fatty acids.

Therefore, increased protein intake could impair beta-oxidation, favoring fat mass deposition [12]. In fact, in the muscle, BCAAs are employed for energy provision, together with short-chain acyl carnitines production. Under physiological conditions, Leu supplementation increases BCKD activity. However, elevated plasma BCAA concentrations can enhance Leu's role as a stimulator of insulin secretion [13], which suppresses the oxidation of fatty acids [14], and may promote the accumulation of fat mass. Some AAs are known to stimulate insulin, glucagon, and growth hormone secretion through different pathways. The liver possesses only a portion of BCKD's catabolic capacity [15], leading to a greater release of BCAAs into the bloodstream compared to other AAs. This explains the substantial variations in plasma BCAA concentrations between HP and LP formulas. Due to BCAAs' influence on fat metabolism, excessive fat deposition may occur, potentially leading to lipotoxicity, insulin resistance and fat storage. This establishes a link between a higher body mass index (BMI) and a higher protein intake [12]. **Supplementary Figure 1** illustrates the relationship between high protein and BCAAs intake with the nutritional-metabolic impact on insulin and fatty acids metabolism.

References

1. Vandenplas, Y.; Broekaert, I.; Domellöf, M.; Indrio, F.; Lapillonne, A.; Pienar, C.; Ribes-Koninckx, C.; Shamir, R.; Szajewska, H.; Thapar, N.; et al. An ESPGHAN Position Paper on the Diagnosis, Management and Prevention of Cow's Milk Allergy. *J Pediatr Gastroenterol Nutr* **2023**, doi:10.1097/MPG.0000000000003897.
2. DRV Finder Available online: https://multimedia.efsa.europa.eu/drvs/index.htm (accessed on 11 January 2025).
3. Holeček, M. Branched-Chain Amino Acids in Health and Disease: Metabolism, Alterations in Blood Plasma, and as Supplements. *Nutr Metab (Lond)* **2018**, *15*, 33, doi:10.1186/s12986-018-0271-1.
4. Bhagavan, N.V.; Ha, C.-E. Chapter 15 - Protein and Amino Acid Metabolism. In *Essentials of Medical Biochemistry*; Bhagavan, N.V., Ha, C.-E., Eds.; Academic Press: San Diego, 2011; pp. 169–190 ISBN 978-0-12-095461-2.
5. Holecek, M.; Kovarik, M. Alterations in Protein Metabolism and Amino Acid Concentrations in Rats Fed by a High-Protein (Casein-Enriched) Diet - Effect of Starvation. *Food Chem Toxicol* **2011**, *49*, 3336–3342, doi:10.1016/j.fct.2011.09.016.
6. Fernicola, J.; Vyavahare, S.; Gupta, S.K.; Kalwaghe, A.; Kosmac, K.; Davis, A.; Nicholson, M.; Isales, C.M.; Shinde, R.; Fulzele, S. The Role of Branched Chain Ketoacid Dehydrogenase Kinase (BCKDK) in Skeletal Muscle Biology and Pathogenesis. *Int J Mol Sci* **2024**, *25*, 7601, doi:10.3390/ijms25147601.
7. de la O, V.; Zazpe, I.; Ruiz-Canela, M. Effect of Branched-Chain Amino Acid Supplementation, Dietary Intake and Circulating Levels in Cardiometabolic Diseases: An Updated Review. *Curr Opin Clin Nutr Metab Care* **2020**, *23*, 35–50, doi:10.1097/MCO.0000000000000614.
8. Green, C.L.; Lamming, D.W. Regulation of Metabolic Health by Essential Dietary Amino Acids. *Mech Ageing Dev* **2019**, *177*, 186–200, doi:10.1016/j.mad.2018.07.004.
9. Zhenyukh, O.; Civantos, E.; Ruiz-Ortega, M.; Sánchez, M.S.; Vázquez, C.; Peiró, C.; Egido, J.; Mas, S. High Concentration of Branched-Chain Amino Acids Promotes Oxidative Stress, Inflammation and Migration of Human Peripheral Blood Mononuclear Cells via mTORC1 Activation. *Free Radic Biol Med* **2017**, *104*, 165–177, doi:10.1016/j.freeradbiomed.2017.01.009.
10. Bloomgarden, Z. Diabetes and Branched-Chain Amino Acids: What Is the Link? *J Diabetes* **2018**, *10*, 350–352, doi:10.1111/1753-0407.12645.

11. Koletzko, B.; von Kries, R.; Closa, R.; Escribano, J.; Scaglioni, S.; Giovannini, M.; Beyer, J.; Demmelmair, H.; Anton, B.; Gruszfeld, D.; et al. Can Infant Feeding Choices Modulate Later Obesity Risk? *Am J Clin Nutr* **2009**, *89*, 1502S-1508S, doi:10.3945/ajcn.2009.27113D.

12. Kirchberg, F.F.; Harder, U.; Weber, M.; Grote, V.; Demmelmair, H.; Peissner, W.; Rzehak, P.; Xhonneux, A.; Carlier, C.; Ferre, N.; et al. Dietary Protein Intake Affects Amino Acid and Acylcarnitine Metabolism in Infants Aged 6 Months. *J Clin Endocrinol Metab* **2015**, *100*, 149–158, doi:10.1210/jc.2014-3157.

13. Kuhara, T.; Ikeda, S.; Ohneda, A.; Sasaki, Y. Effects of Intravenous Infusion of 17 Amino Acids on the Secretion of GH, Glucagon, and Insulin in Sheep. *Am J Physiol* **1991**, *260*, E21-26, doi:10.1152/ajpendo.1991.260.1.E21.

14. Goichon, A.; Chan, P.; Lecleire, S.; Coquard, A.; Cailleux, A.-F.; Walrand, S.; Lerebours, E.; Vaudry, D.; Déchelotte, P.; Coëffier, M. An Enteral Leucine Supply Modulates Human Duodenal Mucosal Proteome and Decreases the Expression of Enzymes Involved in Fatty Acid Beta-Oxidation. *J Proteomics* **2013**, *78*, 535–544, doi:10.1016/j.jprot.2012.10.024.

15. Brosnan, J.T.; Brosnan, M.E. Branched-Chain Amino Acids: Enzyme and Substrate Regulation. *J Nutr* **2006**, *136*, 207S-11S, doi:10.1093/jn/136.1.207S.

Supplementary Figure S1. Branched-chain amino acid intake and oxidation lead to a nutritional-metabolic impact on fatty acid metabolism and fat mass deposition. Whey proteins, which contain BCAAs, including Leu, have a greater impact on insulin production, whereas casein influences growth hormone (GH) production. Both contribute to IGF-1 release. When the body's capacity to utilize amino acids is exceeded, they undergo oxidation via BCKD, leading to the formation of short-chain acylcarnitines. Specifically, C5 further inhibits fatty acid beta-oxidation, promoting adipose tissue accumulation.

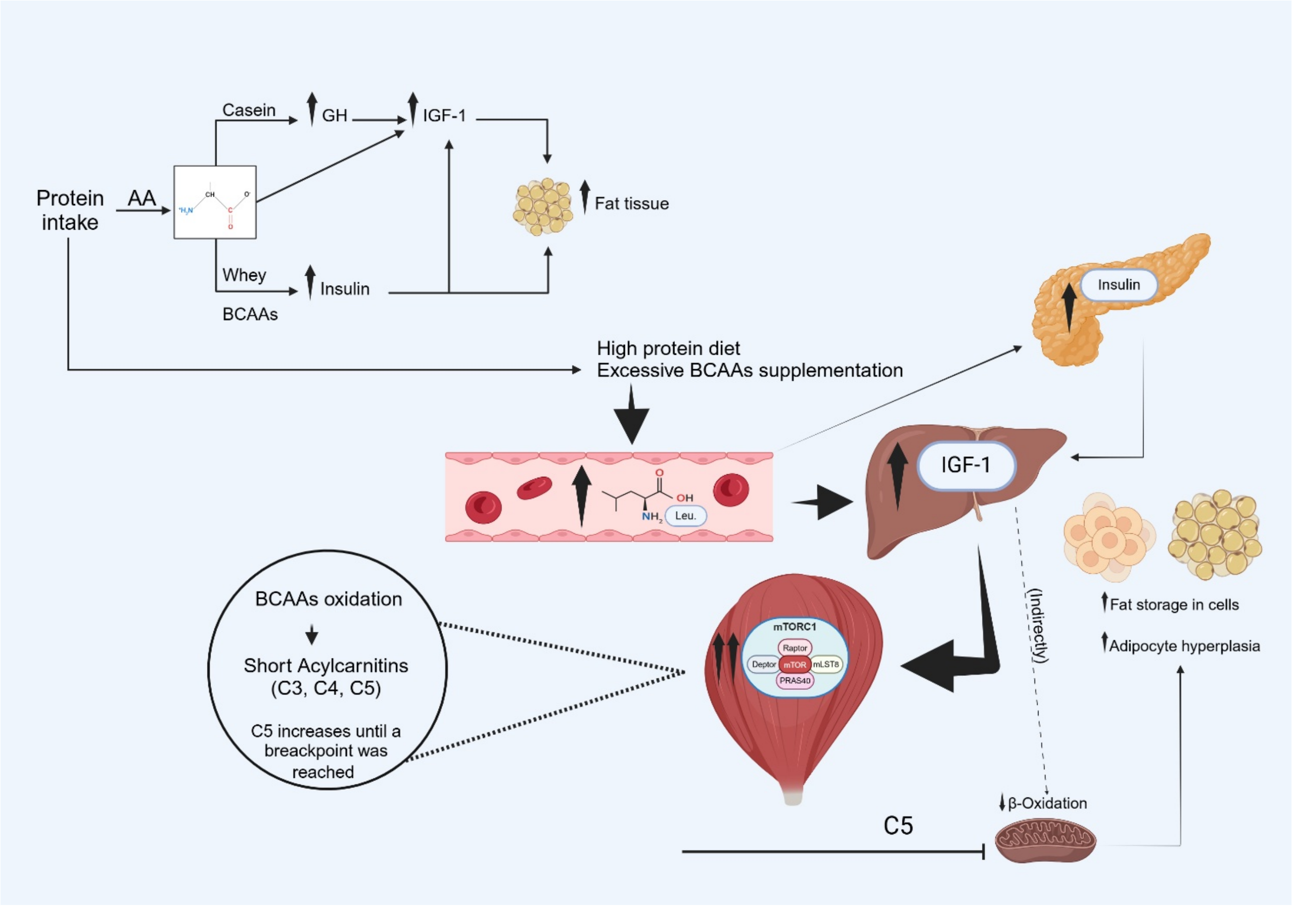

Supplementary Table S1. Protein digestibility according to food sources.

PROTEIN DIGESTIBILITY AND FOOD SOURCES	
Purified or concentrated vegetable proteins (e.g. soy protein, gluten)	**≥ 95%**
Intact vegetable products, such as whole cereals and pulses, protein digestibility is lower	**Around 80-90%**
Most other vegetable proteins (because of the presence of plant cell walls and anti-nutritional factors)	**Around 50-80%**

Supplementary Table S2. PICOS criteria for inclusion of studies.

TOPIC	Narrative question	P - Population	I – Interventions	C - Comparison/Control	O - Outcomes
**Topic 1**<br><br>**Branched chain amino acids (BCAAs): leucine, isoleucine and valine**	Does the supplementation with BCAAs in IMDs improve metabolic stability and biochemical parameters?	Infants, children, adolescents or adults with Urea cycle Disorders, Maple Syrup Urine Disease, Methylmalonic acidemia, Propionic acidemia, Glycogen storage disease due to acid maltase deficiency, defect Isoleucyl-tRNA synthetase defect, Leucyl-tRNA synthetase defect	Supplementation with: leucine, isoleucine and valine	-Pre/post supplementation<br><br>-Natural history<br><br>-Different supplementation doses	Identified through:<br><br>- Biochemical outcomes (correction of secondary deficiencies, reduction of toxic metabolite plasma levels)<br>- Clinical outcomes (improvement in muscle strength and respiratory function, reduction of epithelial damage, improvement in neurological symptoms)
**Topic 2**<br><br>**Aromatic amino acids (AAAs): phenylalanine, tryptophan and tyrosine**	Does the supplementation with AAAs in IMDs improve metabolic stability and biochemical parameters?	Infants, children, adolescents or adults with Phenylketonuria, Phenylalanyl-tRNA synthetase defect	Supplementation with: phenylalanine, tryptophan and tyrosine	-Pre/post supplementation<br><br>-Natural history	Identified through:<br><br>- Biochemical outcomes (reduction of toxic metabolite plasma levels and in brain concentrations, improvement in neurotransmitter homeostasis)<br>- Clinical outcomes (improved neurodevelopment and growth)
**Topic 3**<br><br>**Sulphur amino acids (S-AAs): cystein and methionine**	Does the supplementation with S-AAs in IMDs improve metabolic stability and biochemical parameters?	Infants, children, adolescents or adults with Cystathionine beta-synthase-deficient homocystinuria, Methylmalonic acidemia with homocystinuria, Methylcobalamin	Supplementation with: cysteine and methionine	-Pre/post supplementation<br><br>-Natural history	Identified through:<br><br>- Biochemical outcomes (reduction of toxic metabolite plasma levels, increased excretion of toxic metabolites, increased plasma levels of supplemented amino acid)<br>- Clinical outcomes (improved neurodevelopment and growth, and respiratory function)

		deficiency, Methionyl-tRNA synthetase defect			
**Topic 4**<br><br>**Urea cycle amino acids (UCD-AAs): arginine, citrulline, ornithine**	Does the supplementation with UCD-AAs in IMDs improve metabolic stability and biochemical parameters?	Infants, children, adolescents or adults with Urea Cycle Disorders, Citrin deficiency, Lysinuric protein intolerance, Glutaric Acidemia type 1, Pyridoxine-dependent epilepsy, antiquitin deficiency, X-linked creatine transporter deficiency, Guanidinoacetate methyltransferase deficiency, ALDH18A1-related De Barsy syndrome, Mitochondrial encephalomyopathy, lactic acidosis, and stroke-like episodes syndrome	Supplementation with: arginine, citrulline, ornithine	-Pre/post supplementation<br><br>-Natural history<br><br>-Control group without supplementation	Identified through:<br><br>- Biochemical outcomes (reduction of toxic metabolite plasma levels, increased excretion of toxic metabolites, increased plasma levels of supplemented amino acid)<br>- Clinical outcomes (prevention of stroke-like episodes, improved neurodevelopment outcomes)
**Topic 5**<br><br>**Other essential amino acids (EAAs): threonine and lysine**	Does the supplementation with other EAAs in IMDs improve metabolic stability and biochemical parameters?	Infants, children, adolescents or adults with Ornithine aminotransferase deficiency, Phenylketonuria, Lysinuric protein intolerance	Supplementation with: threonine and lysine	-Pre/post supplementation<br><br>-Natural history	Identified through:<br><br>- Biochemical outcomes (reduction of toxic metabolite plasma levels, increased excretion of toxic metabolites)

**Topic 6**<br><br>**Other non-essential amino acids (non-EAAs): alanine, glycine, glutamine, proline and serine**	Does the supplementation with other non-EAAs in IMDs improve metabolic stability and biochemical parameters?	Infants, children, adolescents or adults with Isovaleric acidemia, X-linked creatine transporter deficiency, Glutamine synthetase deficiency, Ornithine aminotransferase deficiency, ALDH18A1-related De Barsy syndrome, Seryl-tRNA synthetase 1, Neurometabolic disorder due to serine deficiency, 3-Phosphoglycerate dehydrogenase deficiency, Deficiency of phosphoserine aminotransferase, GRIN-related disorders, Glycogen storage disease due to acid maltase deficiency	Supplementation with: alanine, glycine, glutamine, proline and serine	-Pre/post supplementation<br><br>-Natural history<br><br>-Control group without supplementation	Identified through:<br><br>- Biochemical outcomes (reduction of toxic metabolite plasma levels, increased excretion of toxic metabolites, increased plasma levels of supplemented amino acid)<br>- Clinical outcomes (improvement in neurological and cognitive function, in muscle function and in vision)

Supplementary Table S3. Research strategies employed on PubMed/Medline, Scopus and Cochrane library.

**1.**	**Branched chain amino acids (BCAAs): leucine, isoleucine and valine – 147 results**
a)	((leucine AND isoleucine AND valine) AND supplementation) AND (urea cycle disorders) – 11 results
b)	((leucine OR isoleucine OR valine) AND supplementation) AND (urea cycle disorders) – 17 results
c)	((valine OR isoleucine) AND supplementation) AND (maple syrup urine disease) – 24 results
d)	((valine AND isoleucine) AND supplementation) AND (maple syrup urine disease) – 32 results
e)	((valine OR isoleucine) AND supplementation) AND (propionic acidemia) – 20 results
f)	((valine AND isoleucine) AND supplementation) AND (propionic acidemia) – 14 results
g)	((valine OR isoleucine) AND supplementation) AND (methylmalonic acidemia) – 14 results
h)	((valine AND isoleucine) AND supplementation) AND (methylmalonic acidemia) – 13 results
i)	((leucine OR isoleucine OR valine) AND supplementation) AND (glycogen storage disease due to acid maltase deficiency OR pompe disease) – 2 results
j)	((leucine AND isoleucine AND valine) AND supplementation) AND (glycogen storage disease due to acid maltase deficiency OR pompe disease) – 0 results
**2.**	**Aromatic amino acids (AAAs): phenylalanine, tryptophan and tyrosine – 128 results**
a)	(tyrosine supplementation) AND (Phenylketonuria) – 100 results
b)	(tryptophan supplementation) AND (Phenylketonuria) – 24 results
c)	(phenylalanine supplementation) AND (Phenylalanyl-tRNA synthetase) – 4 results
**3.**	**Sulphur amino acids (S-AAs): cystein and methionine – 39 results**
a)	(cysteine supplementation) AND (cystathionine beta-synthase-deficient homocystinuria) – 3 results
b)	(methionine supplementation) AND (methylmalonic acidemia with homocystinuria) – 3 results
c)	(methionine supplementation) AND (methylcobalamin deficiency) – 20 results
d)	(methionine supplementation) AND (methionyl-tRNA synthetase) – 13 results
**4.**	**Urea cycle amino acids (UCD-AAs): arginine, citrulline, ornithine – 261 results**
a)	((arginine AND citrulline) AND supplementation) AND (urea cycle disorders) – 41 results
b)	((arginine OR citrulline) AND supplementation) AND (urea cycle disorders) – 99 results
c)	(arginine supplementation) AND (citrin deficiency)) – 5 results

d)	(citrulline supplementation) AND (lysinuric protein intolerance) – 18 results
e)	(arginine supplementation) AND (glutaric acidemia type 1) – 6 results
f)	(arginine supplementation) AND ((pyridoxine-dependent epilepsy) OR (antiquitin deficiency)) – 24 results
g)	(arginine supplementation) AND (x-linked creatine transporter deficiency) – 14 results
h)	((arginine AND citrulline AND ornithine) AND supplementation) AND ((ALDH18A1-related De Barsy syndrome) OR (P5CS deficit)) – 0 results
i)	((arginine OR citrulline OR ornithine) AND supplementation) AND ((ALDH18A1-related De Barsy syndrome) OR (P5CS deficit)) – 0 results
j)	(ornithine supplementation) AND (guanidinoacetate methyltransferase deficiency) – 20 results
k)	(arginine supplementation) AND (mitochondrial encephalomyopathy, lactic acidosis, and stroke-like episodes syndrome) – 20 results
l)	(citrulline supplementation) AND (mitochondrial encephalomyopathy, lactic acidosis, and stroke-like episodes syndrome) – 14 results
**5.**	**Other essential amino acids (EAAs): threonine and lysine – 44 results**
a)	(lysine supplementation) AND (ornithine aminotransferase deficiency) – 11 results
b)	(threonine supplementation) AND (phenylketonuria) – 4 results
c)	(lysine supplementation) AND (lysinuric protein intolerance) – 29 results
**6.**	**Other non-essential amino acids (non-EAAs): alanine, glycine, glutamine, proline and serine– 115 results**
a)	(glycine supplementation) AND (isovaleric acidemia) – 19 results
b)	(glycine supplementation) AND (x-linked creatine transporter deficiency) – 14 results
c)	(glutamine supplementation) AND (glutamine synthetase deficiency) – 39 results
d)	(proline supplementation) AND (ornithine aminotransferase deficiency) – 4 results
e)	(proline supplementation) AND (ALDH18A1-related De Barsy syndrome) OR (P5CS deficit) – 24 results
f)	(serine supplementation) AND (seryl-tRNA synthetase) – 1 result
g)	(glycine supplementation) AND (neurometabolic disorder due to serine deficiency) – 2 results
h)	(glycine supplementation) AND (deficiency of phosphoserine aminotransferase) – 7 results
i)	(serine supplementation) AND (GRIN disorders) – 0 results

j) (alanine supplementation) AND (glycogen storage disease due to acid maltase deficiency OR pompe disease) – 5 results

Supplementary Table S4. A summary table of the main aetiological and therapeutic characteristics of each IMD is included in this review.

IMD	*Main etiological characteristics and dietary management*
**Phenylketonuria (PKU)**	PKU is due to a deficiency in the enzyme phenylalanine hydroxylase (PAH), leading to the accumulation of Phenylalanine and impacting brain development and functions. The treatment consists of a low-Phe diet but also pharmacological treatment can effective for some patients.
**Tyrosinemia type 1**	It is caused by an enzyme defect of fumarylacetoacetate hydrolase (FAH), leading to the accumulation of tyrosine and its toxic metabolites, such as succinyl acetone (SA). A low Phenylalanine and Tyrosine diet is of utmost importance in reducing the risk of complications. The primary pharmacological treatment involves the use of nitisinone (NTBC).
**Maple Syrup Urine Disease (MSUD)**	MSUD is caused by homozygous or compound heterozygous mutations in one or more subunits of the mitochondrial branched-chain α-ketoacid dehydrogenase (BCKAD) complex. Patients must follow a low-natural protein diet to regulate and minimize the intake of branched-chain amino acids while ensuring adequate levels of protein, fluid, and energy for optimal growth and development. The tolerable levels of BCAAs depend on age, weight, and BCAAs concentration in the blood.
**Glutaric Acidemia type 1 (GA1)**	GA1 is caused by autosomal recessive mutations in the GCDH gene, encoding glutaryl-CoA dehydrogenase, a mitochondrial enzyme that plays a role in the degradation of glutaryl-CoA to crotonyl-CoA. The accumulation of glutaric acid is toxic to the brain. Dietary treatment should consist of a low-lysine and tryptophan diet, as these AA is the precursor of glutaric acid and 3-hydroxyglutaric acid synthesis, and carnitine supplementation.
**Homocystinuria (HCU)**	HCU is caused by the deficiency of cystathionine β-synthase, resulting in an abnormal accumulation of homocysteine and its metabolites in the blood and urine. Individuals who do not respond to vitamin B6 require a methionine-restricted diet through a low methionine intake. Treatment involves vitamin B6 supplementation and often includes betaine.
**Methylmalonic acidemia (MMA)**	MMA is caused by a deficiency of methyl malonyl-CoA mutase, a vitamin B12-dependent mitochondrial enzyme that catalyzes the conversion of methyl malonyl-CoA to succinyl-CoA. The mainstay of the long-term treatment is a low Threonine, Methionine, Valine, and Isoleucine intake, precursors to methylmalonyl-CoA. Prolonged fasting should be prevented to avoid the release of propiogenic odd chain fatty acids derived from lipolysis. Vitamin B12 supplements for responders and carnitine can also be employed.
**Propionic acidemia (PA)**	PA is caused by a deficiency of propionyl-CoA carboxylase, a mitochondrial biotin-dependent enzyme which converts propionyl-CoA to methyl malonyl-CoA. The mainstay of the long-term treatment is a low Threonine, Methionine, Valine, and Isoleucine intake. Moreover, prolonged fasting should be prevented to avoid the release of odd chain fatty acids derived from lipolysis. Carnitine and, for some patients, biotin can also be employed.
**Isovaleric Acidemia (IVA)**	IVA is due to a deficiency of isovaleryl-CoA dehydrogenase, on the leucine catabolic pathway. The mainstay of treatment for IVA involves dietary management to restrict leucine intake. The pharmacological treatment involves glycine and carnitine supplementation.
**Urea Cycle Disorders (UCDs)**	UCDs are inborn errors of nitrogen detoxification/arginine synthesis due to defects in the urea cycle enzymes. Treatment depends on the specific enzymatic defect and includes a combination of pharmacological agents, a low natural protein diet, and appropriate nutritional supplementations (i.e., EAAs or BCAAs), to reduce hyperammonemia and/or improve metabolic stability. UCDs include N-acetylglutamate synthase (NAGS) deficiency, carbamoylphosphate synthetase I (CPS1) deficiency, ornithine transcarbamylase (OTC) deficiency, argininosuccinate synthase 1 (ASS1) deficiency, citrin deficiency, argininosuccinic lyase (ASL) deficiency, arginase (ARG) deficiency and ornithine translocase deficiency.
**Ornithine aminotransferase deficiency (OAT)**	Ornithine aminotransferase deficiency is an inborn error of ornithine metabolism, caused by decreased activity of the enzyme ornithine aminotransferase. It is characterized by poor vision progressing to blindness. In order to reduce the high levels of ornithine in the blood, a diet with restriction in arginine has been used.
**Citrin Deficiency (CD)**	CD is a genetic metabolic disorder caused by mutations in the SLC25A13 gene, which encodes the citrin protein. A diet rich in protein, fats and MCT and low in carbohydrates is recommended.
**ARS deficiencies**	The terms IARS (Isoleucyl-tRNA Synthetase), LARS (Leucyl-tRNA Synthetase), FARS (Phenylalanyl-tRNA Synthetase), MARS (Methionyl-tRNA Synthetase), and SARS (Seryl-tRNA Synthetase) refer to a group of aminoacyl-tRNA synthetases, enzymes critical in the protein synthesis process.
**Pompe Disease**	Also known as Glycogen Storage Disease Type II, it is an inherited lysosomal storage disorder caused by a deficiency of the enzyme acid alpha-glucosidase (GAA). The primary treatment for Pompe disease is enzyme replacement therapy (ERT).
**Lysinuric Protein**	It is a rare autosomal recessive metabolic disorder caused by mutations in the SLC7A7 gene, which encodes the y+LAT1 amino acid transporter, crucial for the

**intolerance (LPI)**	absorption of lysine, arginine, and ornithine. Treatment focuses on symptom management and preventing complications, with a low-protein diet, citrulline supplementation and ammonia control.
**Pyridoxine-dependent epilepsy (PDE) and antiquitin deficiency (ATQ)**	PDE and ATQ, primarily caused by mutations in the ALDH7A1 gene, are both rare neurological disorders involving lysine degradation in the brain. The mainstay of treatment is supplementation with pyridoxine.
**X-linked creatine transporter deficiency (CRTR-D)**	It is a genetic X-linked disorder caused by mutations in the SLC6A8 gene. The disorder manifests primarily in males with a range of neurological and developmental issues.
**Guanidinoacetate methyltransferase deficiency (GAMT)**	GAMT, caused by mutations in the GAMT gene, affects creatine synthesis, leading to neurological and developmental issues, leading to decreased creatine production and an accumulation of guanidinoacetate, which can be neurotoxic. Treatment focuses on creatine and ornithine supplementation, and arginine restriction.
**ALDH18A1-related De Barsy syndrome (P5CS deficiency)**	It is caused by mutations in the ALDH18A1 gene, which encodes the P5CS enzyme, involved in proline and ornithine metabolism and thus affecting collagen and connective tissues synthesis, as well as the urea cycle.
**Mitochondrial encephalomyopathy, lactic acidosis, and stroke-like episodes syndrome (MELAS)**	MELAS is a complex mitochondrial disorder affecting the central nervous system and the muscles because of inadequate energy production. Treatment for MELAS is primarily supportive and aims to manage symptoms, for example with metabolic therapy that can include supplements such as coenzyme Q10, vitamins and cofactors that support mitochondrial function.
**Glutamine synthetase deficiency**	It is characterized by a lack of the enzyme glutamine synthetase, playing a critical role in the synthesis of glutamine from glutamate and ammonia. Symptoms are severe and typically present in the neonatal period or early infancy. Treatment can include ammonia scavengers, dialysis and nutritional support.
**Neurometabolic disorder due to serine deficiency**	It is caused by mutations in genes involved in serine biosynthesis, such as *PHGDH, PSAT1,* or *PSPH*, leading to impaired synthesis of L-serine, essential for brain development, neurotransmitter production, and myelin formation. Treatment primarily involves oral supplementation with L-serine, often combined with glycine.
**3-Phosphoglycerate dehydrogenase deficiency (3-PGDH deficiency)**	It is a rare autosomal recessive disorder caused by mutations in the *PHGDH* gene, leading to reduced serine levels, which can result in severe neurological symptoms, including congenital microcephaly, psychomotor retardation, intractable epilepsy, and spasticity.
**Deficiency of phosphoserine aminotransferase (PSAT)**	PSAT deficiency is caused by mutations in the *PSAT1* gene, which encodes phosphoserine aminotransferase, involved in the serine biosynthesis pathway.
**GRIN-related disorders**	They are a group of rare genetic conditions associated with mutations in the GRIN genes, which encode subunits of the N-methyl-D-aspartate (NMDA) receptor, critical component of synaptic transmission and plasticity in the nervous system. Mutations in GRIN2B can lead to either gain-of-function or loss-of-function effects on the NMDA receptor.
